# The use of angiotensin II for the management of distributive shock: expert consensus statements

**DOI:** 10.1186/s44158-024-00186-y

**Published:** 2024-08-16

**Authors:** Giovanni Landoni, Andrea Cortegiani, Elena Bignami, Gennaro De Pascale, Katia Donadello, Abele Donati, Giacomo Grasselli, Fabio Guarracino, Gianpaola Monti, Gianluca Paternoster, Luigi Tritapepe, Massimo Girardis

**Affiliations:** 1 Anesthesia and Intensive Care Department, IRCCS San Raffaele Scientific Institute, Milan, Italy; 2 Vita-Salute San Raffaele University, Milan, Italy; 3 Department of Precision Medicine in Medical, Surgical and Critical Care (Me.Pre.C.C.), University of Palermo, Palermo, Italy; 4 Department of Anesthesia Intensive Care and Emergency, University Hospital Policlinico Paolo Giaccone, Palermo, Italy; 5 Anesthesiology, Critical Care and Pain Medicine Division, Department of Medicine and Surgery, University of Parma, Parma, Italy; 6 Dipartimento di Scienze Biotecnologiche di Base, Cliniche Intensivologiche e Perioperatorie, Università Cattolica del Sacro Cuore, Rome, Italy; 7 Dipartimento di Scienze dell’Emergenza, Anestesiologiche e della Rianimazione, Fondazione Policlinico Universitario A. Gemelli IRCCS, Rome, Italy; 8 Department of Surgery, Dentistry, Gynecology and Pediatrics, University of Verona, Verona, Italy; 9 Anaesthesia and Intensive Care B, University Hospital Integrated Trust of Verona, Verona, Italy; 10 Department of Biomedical Sciences and Public Health, Università Politecnica delle Marche, Ancona, Italy; 11 Anesthesia and Intensive Care, Azienda Ospedaliero Universitaria Delle Marche, Ancona, Italy; 12 Department of Anesthesia, Intensive Care and Emergency, Fondazione IRCCS Ca’ Granda Ospedale Maggiore Policlinico, Milan, Italy; 13 Department of Pathophysiology and Transplantation, University of Milan, Milan, Italy; 14 Department of Anesthesia and Intensive Care, Azienda Ospedaliero Universitaria Pisana, Pisa, Italy; 15 Department of Anesthesia and postsurgical and abdominal transplantation Intensive Care Unit, ASST GOM Niguarda, Milan, Italy; 16 Cardiovascular Anesthesia and ICU San Carlo Hospital, Potenza, Italy; 17 Department of Anesthesia and Intensive Care, Sapienza University of Rome, Rome, Italy; 18 Department of Anesthesia and Intensive Care, San Camillo-Forlanini Hospital, Rome, Italy; 19 Department of Anesthesia and Intensive Care, University Hospital of Modena, Modena, Italy; 20 University of Modena and Reggio Emilia, Reggio Emilia, Italy

**Keywords:** Angiotensin II, Refractory distributive shock, Vasopressors, Non-catecholaminergic agents, Consensus statements

## Abstract

**Background:**

Despite the growing body of evidence supporting the use of angiotensin II (ATII) in distributive shock, its integration into existing treatment algorithms requires careful consideration of factors related to patient comorbidities, hemodynamic parameters, cost-effectiveness, and risk–benefit balance. Moreover, several questions regarding its use in clinical practice warrant further investigations. To address these challenges, a group of Italian intensive care specialists (the panel) developed a consensus process using a modified Delphi technique.

**Methods:**

The panel defined five clinical questions during an online scoping workshop and then provided a short list of statements related to each clinical question based on literature review and clinical experience. A total of 20 statements were collected. Two coordinators screened and selected the final list of statements to be included in the online survey, which consisted of 17 statements. The consensus was reached when ≥ 75% of respondents assigned a score within the 3-point range of 1–3 (disagreement) or 7–9 (agreement).

**Results:**

Overall, a consensus on agreement was reached on 13 statements defining the existing gaps in scientific evidence, the possibility of evaluating the addition of drugs with different mechanisms of action for the treatment of refractory shock, the utility of ATII in reducing the catecholamine requirements in the treatment of vasopressor-resistant septic shock, and the effectiveness of ATII in treating patients in whom angiotensin-converting enzyme activity is reduced or pharmacologically blocked. It was widely shared that renin concentration can be used to identify patients who most likely benefit from ATII to restore vascular tone. Thus, the patients who might benefit most from using ATII were defined. Lastly, some potential barriers to the use of ATII were described.

**Conclusions:**

ATII was recognized as a useful treatment to reduce catecholamine requirements in treating vasopressor-resistant septic shock. At the same time, the need for additional clinical trials to further elucidate the efficacy and safety of ATII, as well as investigations into potential mechanisms of action and optimization of treatment protocols in patients with refractory distributive shock, emerged.

## Background

Distributive shock represents a critical state of cardiovascular dysfunction characterized by widespread vasodilation, impaired tissue perfusion, and consequent organ dysfunction [1–3]. This type of shock is the most common among critically ill patients [4] and poses significant challenges in clinical management, carrying a high mortality rate [2].

The etiology of distributive shock encompasses various pathological processes, including sepsis, anaphylaxis, neurogenic injury, and severe inflammatory responses. Despite the diverse triggers, the hallmark of distributive shock remains the inappropriate distribution of blood flow due to vasoplegia, capillary leak syndrome, and microcirculation impairment, resulting in systemic hypoperfusion [5].

Distributive shock treatment focuses on restoring oxygen delivery to tissues and organs. In this context, timely vasopressor administration and fluid resuscitation are the cornerstones of treatment algorithms [2]. Among vasopressors, norepinephrine, as a first-line agent and, secondly, vasopressin are recommended by evidence-based guidelines [2]. However, both drugs may have substantial harmful effects, such as bowel ischemia, excessive peripheral vasoconstriction, and cardiac stunning [6]. These adverse consequences usually occur at high doses of vasopressors in patients with refractory shock, which is characterized by persistent hypotension despite receiving a baseline dose of norepinephrine base, or equivalent, above 0.5 mcg/kg/min [7]. The aforementioned factors collectively underscore the necessity to develop innovative therapeutic approaches for distributive shock. Given the current lack of guidelines or established protocols for non-catecholaminergic support, particularly in patients with refractory shock, further exploration in this area is justified.

Recently, the vasopressor angiotensin II (ATII), a key effector peptide in the renin–angiotensin–aldosterone system [8], was approved in the USA (December 2017) and in the European Union (August 2019) for use as a second-line vasopressor for the treatment of catecholamine-refractory distributive shock. The pharmacokinetic/pharmacodynamic (PK/PD) profile of AT II makes it an effective agent for rapid and controlled increase in blood pressure in critically ill patients, and quick onset and short duration of action allow for precise titration, which is crucial in the dynamic and often unstable clinical settings where it is used. In particular, once administered intravenously, AT II rapidly distributes in the extracellular fluid and exerts its effects almost immediately. It is primarily metabolized by peptidases present in the blood and tissues, converting it to inactive metabolites. The half-life is very short, typically less than a minute, which necessitates continuous infusion to maintain its therapeutic effect [Baker 2018].

The mechanisms underlying the therapeutic activity of exogenous ATII in distributive shock are multifaceted [9]. By acting on angiotensin receptors within the vascular smooth muscle and endothelium, ATII promotes vasoconstriction, thereby enhancing systemic vascular resistance and restoring arterial pressure [8, 10]. Furthermore, ATII-mediated sympathetic nervous system activation augments cardiac output and improves tissue perfusion [10].

Clinical studies evaluating the use of ATII in critically ill patients with distributive shock reported promising results, with improvements in hemodynamic parameters, reduction in vasopressor requirements, and enhanced organ perfusion [11–15]. In particular, the intravenous Angiotensin II for the Treatment of High-Output Shock (ATHOS) pilot study showed that ATII was an effective “rescue” vasopressor agent in patients with distributive shock who required treatment with multiple vasopressors [12]. In the Angiotensin II for the Treatment of High-Output Shock 3 (ATHOS-3) trial, the largest trial of human ATII in patients with vasodilatory shock, the addition of ATII to background vasopressors improved blood pressure in patients with catecholamine-resistant vasodilatory shock [13]. In addition, ATII therapy appears to have a favorable safety profile, with minimal adverse effects reported in clinical trials.

Despite the growing body of evidence supporting the use of ATII in distributive shock, its integration into existing treatment algorithms requires careful consideration of patient comorbidities, hemodynamic parameters, cost-effectiveness, and risk–benefit balance. Moreover, several questions regarding its use in clinical practice, such as patient selection and long-term outcomes, warrant further investigations [16–19].

To address these challenges, a group of Italian intensive care specialists with extensive clinical and scientific expertise developed a consensus process using a modified Delphi technique. The main aim of this process was to create consensus statements regarding the use of ATII in distributive shock management. This article discloses and critically examines the outcomes of this initiative.

## Methodology

### Project workflow

This project was conducted to reach a consensus on key aspects of using ATII to manage distributive shock, according to a modified Delphi method, and supervised by a panel member with expertise as a methodologist (A. C.). The panel comprised two project coordinators (M. G., G. L.) and nine panelists selected according to clinical and scientific experience in managing patients with distributive shock.

**Fig. 1:**
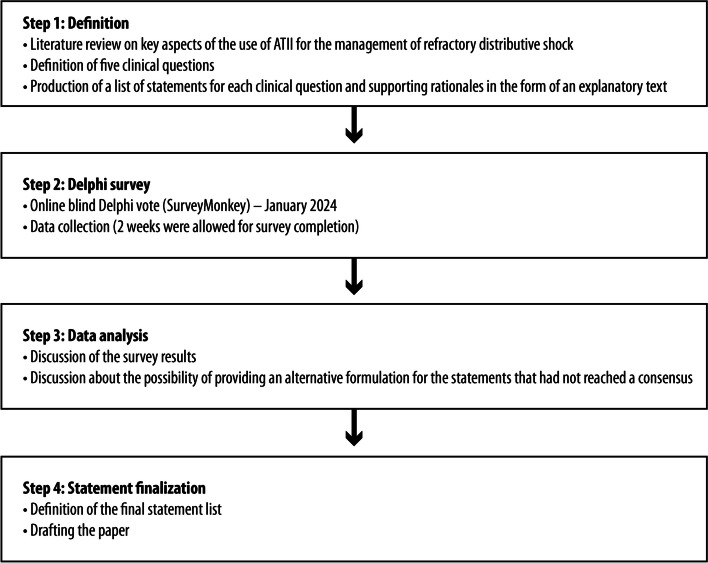
Project workflow

The panel defined five clinical questions (see the following paragraph) during an online scoping workshop. Following the definition of the questions, the coordinators assigned work on each clinical question to a pair of experts within the panel, who were required to provide a short list of statements for the assigned clinical question and supporting rationales in the form of an explanatory text. The entire panel participated in an online blind Delphi vote. The methodology dictated a maximum of two possible voting rounds. The project workflow is reported in Fig. 1.

### Clinical questions and Delphi questionnaire

The panel defined five clinical questions of relevant interest to reach a consensus:

1. In terms of scientific evidence, what gaps exist today?
2. What protocols/regimens/guidelines apply to the treatment of patients with refractory distributive shock hypotension?
3. What are the main pathophysiological advantages of ATII over other available treatments?
4. Which patient could benefit most from using ATII?
5. What are the potential barriers to the use of ATII?

Members of the panel provided a short list of statements related to the assigned clinical question based on a literature review conducted through a comprehensive search of the PubMed/MEDLINE database, as well as clinical experience. The search strategy utilized a combination of keywords related to the use of ATII for the management of distributive shock (e.g., refractory distributive shock hypotension AND ATII, refractory distributive shock hypotension AND management, refractory distributive shock hypotension AND guidelines, ATII AND catecholamine). Inclusion criteria encompassed all types of articles published in English from inception to April 2024. References of the articles were screened by title and abstracts to identify relevant information on these topics. Further full-text screening was done for previously published articles to identify gaps in the selected literature and elaborate on the importance of this topic comprehensively.

A total of 20 statements were collected. The coordinators screened and selected the final list of statements to be included in the online survey, which consisted of 17 statements.

### Consensus criteria

Opinions were expressed using an ordinal Likert scale, according to the RAND-UCLA method (minimum score, 1 = completely disagree; maximum score, 9 = completely agree). This scale was divided into three sections: 1–3 implied refusal/disagreement (“inappropriate”), 4–6 implied “uncertainty,” and 7–9 implied agreement/support (“appropriateness”) [20]. Consensus was reached when 75% or more of the respondents assigned a score within the 3-point range of 1–3 (disagreement) or 7–9 (agreement), which rejected or accepted the statement, respectively. In the absence of an agreement or disagreement score, the result was classified as “uncertainty.”

### Data collection and analysis

The Delphi voting was conducted in January 2024. In the first round, expert panelists responded to an online questionnaire (SurveyMonkey software) and were offered the possibility of adding their opinions using an open text box. The research assistance team (see the “Acknowledgements” section), with the input of the coordinators, evaluated and presented the results of the first round of voting in the form of bar graphs to facilitate discussion during an online meeting. During this meeting, the panel openly discussed the possibility of reformulating statements for which no agreement was reached. All data were analyzed using descriptive statistics provided by SurveyMonkey software.

## Results

**Fig. 2:**
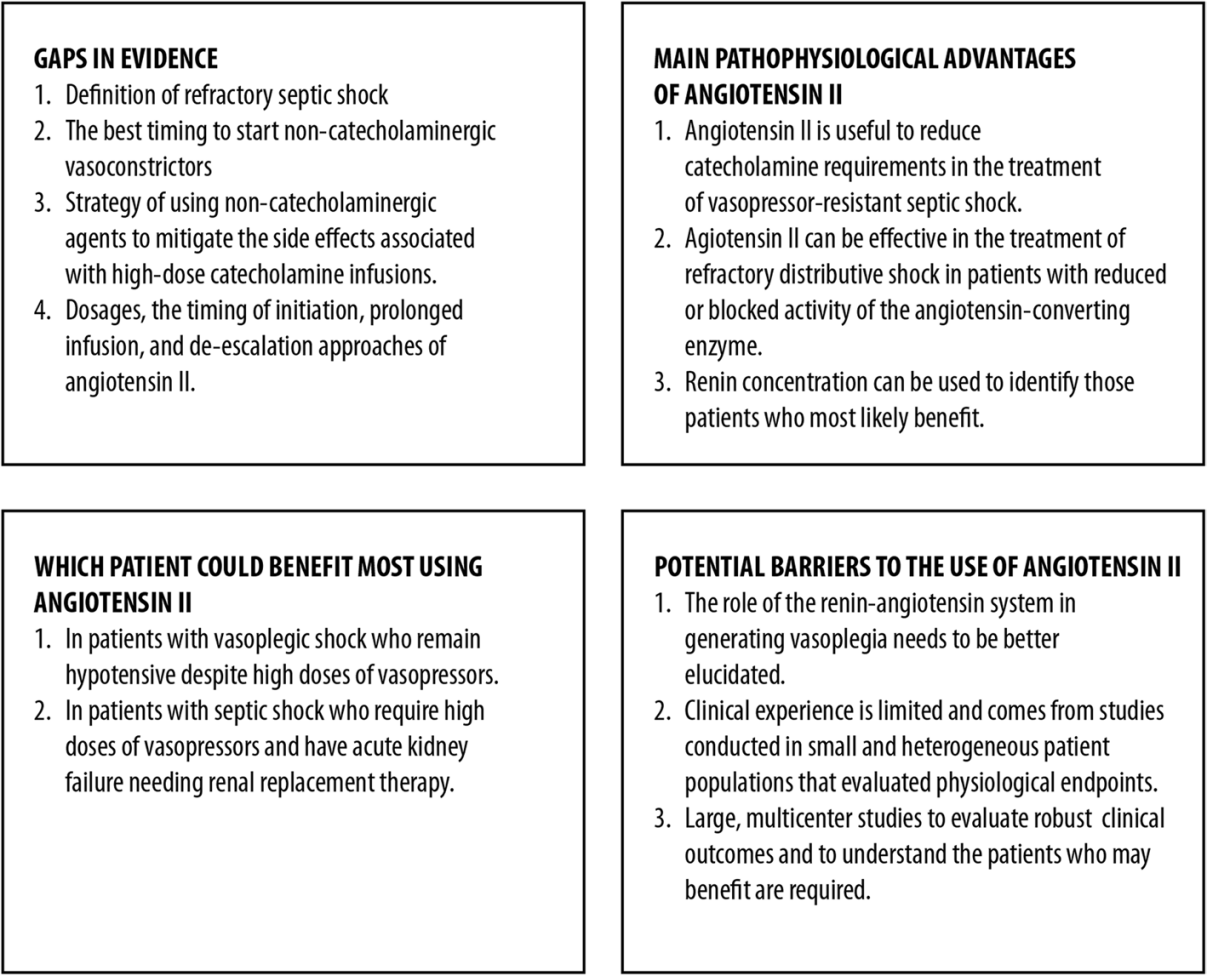
Use of angiotensin II in the management of distributive shock

The final voting results are presented in Table 1. Overall, consensus on the agreement was reached for 13 of the 17 statements (76%) (Fig. 2). Uncertainty was reported for statements 2, 6, 7, and 14 (gray statements in Table 1). Since none of the statements was rejected, the consensus process ended after the first round of voting.

**Table 1:**
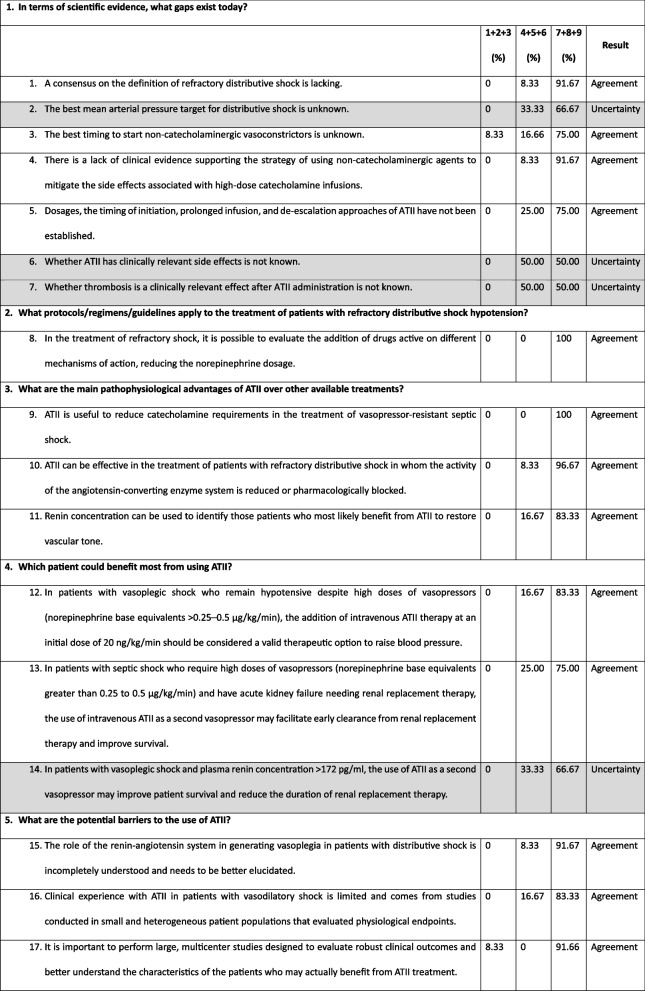
Results of the Delphi panel voting on the use of ATII for the management of distributive shock

## Discussion

To reach a consensus on the key aspects of the use of ATII for the management of refractory distributive shock, an expert panel produced 17 statements in relation to 5 clinical questions, summarizing the direct clinical experience and available literature evidence. Overall, a consensus on agreement was reached for 13 statements (Table 1; Fig. 2).

1. In terms of scientific evidence, what gaps exist today?

The panel widely agreed that a consensus on the definition of refractory distributive shock is lacking, and that the best timing to start non-catecholaminergic vasoconstrictors is still unknown (statements 1 and 2: 91.67% and 75.00% agreement, respectively).

Vasopressors and catecholamines, mainly with alpha-1 adrenergic (norepinephrine, epinephrine), are commonly used to increase mean arterial pressure values during distributive shock. However, they have several side effects. Despite this evidence, treatment with norepinephrine is recommended as the first approach; the lack of clinical evidence supporting the use of non-catecholaminergic agents to mitigate the side effects of high-dose catecholamine was reported as a current gap (statement 4; 91.67% agreement). Given the sound pathophysiological rationale for the use of non-catecholaminergic therapies, the panel agreed that a more comprehensive understanding of the mechanisms underlying distributive shock could lead to a consistent application of a non-catecholaminergic strategy for managing this clinical issue.

Definitions of dosages, timing of initiation, and approaches to prolonged infusion and de-escalation of ATII represent a current knowledge gap (statement 5; 75.00% agreement). Indeed, available data from a few randomized controlled trials showed unclear beneficial effects on survival [13, 19]. In these studies, ATII was administered at a maximum dosage of 80 ng/kg/min during the first 3 h [19].

The panel did not unanimously agree on the lack of an optimal mean arterial pressure target for distributive shock (statement 2, 66.67% agreement) and on the fact the side effects of ATII are not known, particularly with regard to thrombosis (statements 6 and 7, 50.00% agreement). Thus, these areas are considered uncertain or ambiguous.

2. What protocols/regimens/guidelines apply to the treatment of patients with refractory distributive shock hypotension?

The panel agreed on the possibility of evaluating the addition of drugs with different mechanisms of action, thus reducing norepinephrine dosage, for the treatment of refractory distributive shock (statement 8; 100% agreement). This suggestion is in line with the Surviving Sepsis Campaign guidelines, which recommend adding vasopressin instead of increasing the dosage of catecholamines in patients with refractory shock [2]. Other studies, such as the VASST and VANISH trials, have demonstrated the efficacy of vasopressin in reducing the need for norepinephrine (catecholamine-sparing effect) [16, 21].

3. What are the main pathophysiological advantages of ATII over other available treatments?

The panel unanimously agreed on the utility of ATII in reducing the catecholamine requirements in the treatment of vasopressor-resistant septic shock (statement 9; 100% agreement) and on its effectiveness in treating patients in whom angiotensin-converting enzyme activity is reduced or pharmacologically blocked (statement 10; 96.76% agreement). This consensus is based on the results of a subanalysis of the ATHOS-3 study, which showed that populations whose mean arterial pressure increased with ATII administration were those with functional angiotensin-converting enzyme deficiency [22].

It was widely shared that renin concentration can be used to identify patients who most likely benefit from ATII administration to restore vascular tone (statement 11; 83.33% agreement). This suggestion aligns with the results of the study by Bellomo et al., involving patients with baseline renin concentrations higher than normal (normal range, 2.13–58.78 pg/ml) in 194 out of 255 cases (76%; mean renin concentration of 172.7 pg/ml; *IQR*: 60.7–440.6 pg/ml, approximately three times higher than normal) [23]. In this study, renin concentrations positively correlated with ATI/II ratios (*r* = 0.39; *p* = 0.001). Three hours after the initiation of ATII infusion, there was a 54% reduction (*IQR*: 37.9–66.5%) in renin concentrations, compared with a 14% reduction in placebo-treated patients (*p* = 0.0001) [23]. In patients with renin concentrations above the median of the study population, ATII significantly reduced 28-day mortality compared with placebo-treated patients (51% vs 70%) [23]. In addition, a post hoc analysis of the ATHOS-3 study supported the use of ATII in patients with acute kidney injury and renal replacement therapy [24].

4. Which patient could benefit most from using ATII?

According to the panel opinion, in patients with vasoplegic shock who remain hypotensive despite high doses of vasopressors (norepinephrine base equivalents > 0.25–0.5 μg/kg/min), the addition of intravenous ATII therapy at an initial dose of 20 ng/kg/min should be considered a valid therapeutic option to raise blood pressure (statement 12; 83.33% agreement).

Similarly, the panel suggested that in patients with septic shock who need high doses of vasopressors (norepinephrine base > 0.25 to 0.5 μg/kg/min) and have acute kidney failure requiring renal replacement therapy, the use of intravenous ATII as a second vasopressor may facilitate early clearance from renal replacement therapy and improve survival (statement 13; 75.00% agreement). This may be explained by the ability of ATII to enhance glomerular filtration rates by preferentially narrowing efferent renal arterioles. Accordingly, the results of a post hoc subgroup analysis of the ATHOS-3 trial showed that in patients requiring kidney replacement therapy at randomization, the use of ATII was associated with a statistically significant benefit in 28-day mortality (absolute risk reduction of 23%, *NNT* = 4) and days free from renal replacement therapy [24].

The panel deemed uncertain the role of ATII as a second vasopressor in patients with vasoplegic shock and plasma renin concentration > 172 pg/ml in improving patient survival and reducing the duration of renal replacement therapy (statement 14; 66.67% agreement).

5. What are the potential barriers to the use of ATII?

The expert panel agreed on three main potential barriers to the use of ATII in clinical practice: (a) the incomplete knowledge of the role of renin-angiotensin system in the generation of vasoplegia in patients with distributive shock (statement 15; 91.67% agreement); (b) the limited clinical experience with ATII in patients with vasodilatory shock, which stems mainly from studies conducted in small and heterogeneous patient populations that assessed physiological endpoints (statement 16; 83.33% agreement); and (c) the absence of large, multicenter studies with robust clinical outcomes to better understand the characteristics of patients who may benefit from treatment with ATII (statement 17; 91.66% agreement).

## Conclusions

The expert panel provided 13 consensus statements on the use of ATII in the management of refractory distributive shock. These statements are based on a comprehensive review of available evidence, including clinical trials and observational studies, as well as on daily clinical practice.

Overall, a wide consensus was reached on the opinion that combined treatment with drugs with different mechanisms of action may be effective in reducing norepinephrine dosage in patients with refractory shock. With this aim, ATII was widely recognized as a useful treatment for reducing catecholamine requirements. At the same time, consensus statements revealed the need for additional clinical trials to further elucidate the efficacy and safety of ATII, as well as investigations into potential mechanisms of action and optimization of treatment protocols in patients with refractory distributive shock.

## Data Availability

The datasets generated during and/or analyzed during the current study are available from the corresponding author upon reasonable request.

## Abbreviation

ATII: Angiotensin II
